# A Nova Fronteira do Ultrassom de Point-of-Care: Ultrassom de Pulmão na Cardiopatia Congênita

**DOI:** 10.36660/abc.20260163

**Published:** 2026-04-23

**Authors:** Maria Estefânia Bosco Otto

**Affiliations:** 1 Universidade de Brasilia Brasília DF Brasil Universidade de Brasilia, Brasília, DF – Brasil; 2 Hospital DF Star Brasília DF Brasil Hospital DF Star, Brasília, DF – Brasil

**Keywords:** Ultrassonografia, Sistemas Automatizados de Assistência Junto ao Leito, Pulmão, Edema Pulmonar, Cardiopatias Congênitas, Pediatria

A avaliação da congestão e das alterações pulmonares à beira do leito vive um momento de consolidação conceitual: a ultrassonografia de pulmão (USP) deixou de ser apenas um "adjuvante" e passou a ocupar papel central na identificação do líquido extravascular e padrões de aeração, com implicações diagnósticas, prognósticas e terapêuticas. O artigo de Donmez et al.^1^ discute de forma direta o papel do USP na avaliação de edema pulmonar em crianças com cardiopatia congênita (CC). Em uma coorte retrospectiva de 62 crianças, o estudo observou edema pulmonar em 53% pela USP e em 58% pela radiografia.^1^ Ademais, a presença de edema por USP, se associou a variáveis clinicamente relevantes na CC, como infecções respiratórias recorrentes, baixo peso e níveis elevados de BNP.^1^ Estes achados reforçam que o USP acrescenta informação objetiva e reprodutível ao cuidado de crianças, em um contexto no qual o exame clínico pode ser prejudicado por baixa cooperação e a radiografia realizada à beira-leito pode ter acurácia reduzida por limitações de posicionamento e qualidade técnica.

Em adultos com insuficiência cardíaca, a utilidade do USP para congestão pulmonar é sustentada por diversos estudos clínicos que demonstram desempenho diagnóstico elevado e correlação com desfechos. As linhas B refletem aumento de fluido extravascular e, quando presentes de forma difusa, reclassificam o diagnóstico diferencial de dispneia. A comparação entre USP, biomarcadores e avaliação clínica em contexto ambulatorial já mostrou que a carga de linhas B pode detectar congestão subclínica e antecipar a possibilidade de descompensação.^2,3^ Em um cenário agudo, a implementação sistemática do USP melhora a acurácia diagnóstica para insuficiência cardíaca descompensada,^4^ e estudos prognósticos associam congestão detectada ao USP a piores desfechos de curto e longo prazo.^5^ Revisões e perspectivas em cardiologia reforçam o USP como novo método diagnóstico por ser rápido, reprodutível e de baixo custo.^6^

A transposição desse raciocínio para pediatria é atraente, mas exige atenção às especificidades da CC. Em crianças com CC, o edema pulmonar frequentemente reflete hiperfluxo, com desequilíbrio hidrostático-oncótico e disfunção alvéolo-capilar—mecanismos que diferem do padrão típico do adulto de aumento das pressões de enchimento e congestão retrógrada. Dessa forma, estudos em pacientes pediátricos são necessários para consolidar o uso do USP.

Na análise de estudos como o de Wu et al., o qual avaliou o hiperfluxo pulmonar em crianças com CC, se percebe que o USP mostrou desempenho diagnóstico superior ao RX de tórax. O USP demonstrou sensibilidade e especificidade muito altas, com acurácia global de 96%, marcadamente maior do que a radiografia.^7^ Em outras palavras, o USP se apresenta como uma ferramenta não invasiva, reprodutível, e que pode facilmente ser utilizada à beira do leito.

Outro eixo de evidência pediátrica vem do centro cirúrgico: em ensaio clínico randomizado com crianças portadoras de CC sob anestesia geral, Wu et al. observaram que regiões inferoposteriores concentram maior ocorrência de atelectasia ao USP e são mais eficientes para monitorar mudanças após intervenção com uso de pressão positiva.^8^ O estudo é relevante por dois motivos: (i) demonstra que o USP não é apenas "diagnóstico", mas também um método de monitorização dinâmica de recrutamento/aeração; e (ii) sugere racionalização do exame, reduzindo tempo ao focar áreas com maior sensibilidade para mudança—uma necessidade real em pediatria, na qual o tempo de exame e a manipulação do paciente importam.

Ao comentar o artigo de Donmez et al.,^1^ é importante reconhecer seu papel como peça relevante na consolidação do USP no cuidado de crianças com CC: ao demonstrar alta frequência de edema por USP e sua associação com marcadores clínicos de gravidade, o estudo contribui para aproximar a prática pediátrica de uma ferramenta já bem incorporada na cardiologia do adulto.^2-6^ Em pediatria, entretanto, a heterogeneidade das cardiopatias, os mecanismos distintos de edema e as limitações técnicas dos métodos tradicionais à beira do leito reforçam a necessidade de padronização. Assim, o avanço do campo depende agora de estudos prospectivos, idealmente multicêntricos, que definam protocolos por faixa etária e cenário assistencial (enfermaria, emergência, perioperatório), estabeleçam critérios operacionais (regiões de varredura, escores, pontos de corte), avaliem reprodutibilidade interobservador e, sobretudo, testem se estratégias guiadas pelo USP modificam conduta e desfechos. Nesse sentido, o artigo de Donmez et al.^1^ representa um passo determinante: não apenas soma evidências, mas aponta o caminho para que o USP se torne método amplamente aplicável, comparável entre serviços e clinicamente acionável na CC com potencial de expansão para a prática pediátrica em geral.

## References

[1] 1 Donmez YN, Bako D, Alpat S, Giray D, Epcacan S. Improving Pulmonary Edema Assessment in Pediatric Congenital Heart Disease: The Role of Lung Ultrasonography. Arq Bras Cardiol. 2026; 123(3):e20250322. doi: 10.36660/abc.20250322i. PMC13128202 42138860

[2] 2 Otto MEB, Esmanhoto VA, Correia EB, Murta ACS, Brusck LVR, Vilela AA, et al. Pulmonary Congestion in Heart Failure with Reduced Ejection Fraction: Comparison between Lung Ultrasound and Remote Dielectric Sensing. ABC Imagem Cardiovasc. 2023;36(2):e20230027. doi: 10.36660/abcimg.20230027.

[3] 3 Miglioranza MH, Gargani L, Sant’Anna RT, Rover MM, Martins VM, Mantovani A, et al. Lung Ultrasound for the Evaluation of Pulmonary Congestion in Outpatients: A Comparison with Clinical Assessment, Natriuretic Peptides, and Echocardiography. JACC Cardiovasc Imaging. 2013;6(11):1141-51. doi: 10.1016/j.jcmg.2013.08.004. 10.1016/j.jcmg.2013.08.004 24094830

[4] 4 Pivetta E, Goffi A, Lupia E, Tizzani M, Porrino G, Ferreri E, et al. Lung Ultrasound-Implemented Diagnosis of Acute Decompensated Heart Failure in the ED: A SIMEU Multicenter Study. Chest. 2015;148(1):202-10. doi: 10.1378/chest.14-2608. 10.1378/chest.14-2608 25654562

[5] 5 Platz E, Campbell RT, Claggett B, Lewis EF, Groarke JD, Docherty KF, et al. Lung Ultrasound in Acute Heart Failure: Prevalence of Pulmonary Congestion and Short- and Long-Term Outcomes. JACC Heart Fail. 2019;7(10):849-58. doi: 10.1016/j.jchf.2019.07.008. 10.1016/j.jchf.2019.07.008 PMC8409324 31582107

[6] 6 Picano E, Pellikka PA. Ultrasound of Extravascular Lung Water: A New Standard for Pulmonary Congestion. Eur Heart J. 2016;37(27):2097-104. doi: 10.1093/eurheartj/ehw164. 10.1093/eurheartj/ehw164 PMC4946750 27174289

[7] 7 Wu L, Hou Q, Lu Y, Bai J, Sun L, Huang Y, et al. Feasibility of Lung Ultrasound to Assess Pulmonary Overflow in Congenital Heart Disease Children. Pediatr Pulmonol. 2018;53(11):1525-32. doi: 10.1002/ppul.24169. 10.1002/ppul.24169 30251402

[8] 8 Wu L, Hou Q, Bai J, Zhang J, Sun L, Tan R, et al. Modified Lung Ultrasound Examinations in Assessment and Monitoring of Positive End-Expiratory Pressure-Induced Lung Reaeration in Young Children with Congenital Heart Disease Under General Anesthesia. Pediatr Crit Care Med. 2019;20(5):442-9. doi: 10.1097/PCC.0000000000001865. 10.1097/PCC.0000000000001865 31058784

