# Supplementary material for: A Nova Fronteira do Ultrassom de Point-of-Care: Ultrassom de Pulmão na Cardiopatia Congênita
Source: Arq Bras Cardiol. 2026 Apr 23;123(3):e20260163. [Article in Portuguese] doi: 10.36660/abc.20260163 (PMC13128215; doi:10.36660/abc.20260163)
Supplement: Translation

# Translation: The New Frontier of Point-of-Care Ultrasound: Lung Ultrasound in Congenital Heart Disease

**DOI:** 10.36660/abc.20260163i

**Authors:** Maria Estefânia Bosco Otto

**Affiliations:** 1 Brasília DF Brazil Universidade de Brasilia, Brasília, DF – Brazil; 2 Brasília DF Brazil Hospital DF Star, Brasília, DF – Brazil

**Keywords:** Ultrasonography, Point-of-Care Systems, Lung, Pulmonary Edema, Congenital Heart Defects, Pediatrics

The bedside assessment of pulmonary congestion is undergoing conceptual consolidation: lung ultrasound (LUS) has evolved from being merely an "adjunct" tool to assuming a central role in the identification of extravascular fluid and aeration patterns, with important diagnostic, prognostic, and therapeutic implications. The article by Donmez et al.^1^ directly discusses the role of LUS in the assessment of pulmonary edema in children with congenital heart disease (CHD). In a retrospective cohort of 62 children, the study observed pulmonary edema in 53% by LUS and in 58% by radiography.^1^ Furthermore, the presence of edema by LUS was associated with clinically relevant variables in the clinical picture, such as recurrent respiratory infections, low weight, and elevated BNP^1^ levels. These findings reinforce that LUS adds objective and reproducible information to the care of children, in a context where clinical examination can be hampered by low cooperation and bedside radiography may have reduced accuracy due to positioning limitations and technical quality.

In adults with heart failure, the usefulness of LUS for pulmonary congestion is supported by several clinical studies demonstrating high diagnostic performance and correlation with outcomes. B-lines reflect increased extravascular fluid and, when diffusely present, reclassify the differential diagnosis of dyspnea. Comparison between LUS, biomarkers, and clinical assessment in an outpatient setting has shown that the burden of B-lines can detect subclinical congestion and anticipate the possibility of decompensation.^2,3^ In an acute setting, the systematic implementation of LUS improves diagnostic accuracy for decompensated heart failure,^4^ and prognostic studies associate congestion detected by LUS with worse short- and long-term outcomes.^5^ Reviews and perspective papers in cardiology have highlighted LUS as a rapid, reproducible, and low-cost diagnostic modality.^6^

The transposition of this reasoning to pediatrics is attractive, but requires attention to the specificities of the CHD. In children with CHD, pulmonary edema frequently reflects hyperflow, with hydrostatic-oncotic imbalance and alveolar-capillary dysfunction—mechanisms that differ from the typical adult pattern of increased filling pressures and retrograde congestion. Therefore, studies in pediatric patients are needed to consolidate the use of the LUS.

In analyzing studies such as that of Wu et al., which evaluated pulmonary hyperflow in children with CHD, it is clear that LUS showed superior diagnostic performance compared to chest X-ray. LUS demonstrated very high sensitivity and specificity, with an overall accuracy of 96%, markedly higher than radiography.^7^ In other words, LUS presents itself as a non-invasive, reproducible tool that can easily be used at the bedside.

Another line of pediatric evidence comes from the operating room: in a randomized clinical trial with children with CHD under general anesthesia, Wu et al. observed that inferoposterior regions concentrate a higher occurrence of atelectasis on ultrasound and are more efficient for monitoring changes after intervention with positive pressure.^8^ The study is relevant for two reasons: (i) it demonstrates that ultrasound is not only "diagnostic" but also a method for dynamic monitoring of recruitment/aeration; and (ii) it suggests rationalizing the examination, reducing time by focusing on areas with greater sensitivity to change—a real need in pediatrics, where examination time and patient handling matter.

The article by Donmez et al. should be recognised as a relevant contribution to the consolidation of LUS in the care of children with CHD. By demonstrating a high frequency of ultrasound-detected edema and its association with clinical markers of severity, the study helps advance the incorporation of a tool already well established in adult cardiology into pediatric practice.^2-6^ In CHD, however, the heterogeneity of heart diseases, the distinct mechanisms of edema, and the technical limitations of traditional bedside methods reinforce the need for standardization. Thus, progress in the field now depends on prospective, ideally multicentre, studies designed to establish protocols according to age group and clinical setting (ward, emergency department, and perioperative care). Establish operational criteria (scan regions, scores, cut-off points), evaluate interobserver reproducibility, and, above all, test whether ultrasound scan-guided strategies modify management and outcomes. In this sense, the article by Donmez et al.^1^ represents a decisive step: it not only adds evidence but also points the way for LUS to become a widely applicable method, comparable across services and clinically actionable in CHD with the potential for expansion into pediatric practice in general.
